# Electrochemical Behavior of Al-B_4_C Metal Matrix Composites in NaCl Solution

**DOI:** 10.3390/ma8095314

**Published:** 2015-09-21

**Authors:** Yu-Mei Han, X.-Grant Chen

**Affiliations:** Department of Applied Sciences, Université du Québec à Chicoutimi, 555 boul. de l’Université, Saguenay, QC G7H 2B1, Canada; E-Mail: xgrant_chen@uqac.ca

**Keywords:** Al-B_4_C metal matrix composite, pitting, galvanic corrosion, oxygen diffusion

## Abstract

Aluminum based metal matrix composites (MMCs) have received considerable attention in the automotive, aerospace and nuclear industries. One of the main challenges using Al-based MMCs is the influence of the reinforcement particles on the corrosion resistance. In the present study, the corrosion behavior of Al-B_4_C MMCs in a 3.5 wt.% NaCl solution were investigated using potentiodynamic polarization (PDP) and electrochemical impedance spectroscopy (EIS) techniques. Results indicated that the corrosion resistance of the composites decreased when increasing the B_4_C volume fraction. Al-B_4_C composite was susceptible to pitting corrosion and two types of pits were observed on the composite surface. The corrosion mechanism of the composite in the NaCl solution was primarily controlled by oxygen diffusion in the solution. In addition, the galvanic couples that formed between Al matrix and B_4_C particles could also be responsible for the lower corrosion resistance of the composites.

## 1. Introduction

Aluminum based metal matrix composites (MMCs) have received considerable attention in the automotive, aerospace and nuclear industries due to their light weight, as well as their superior thermal conductivity, high stiffness and hardness [1,2,3]. The common reinforcements added to commercial MMCs are silicon carbide (SiC), alumina (Al_2_O_3_) and boron carbide (B_4_C). Compared to traditional SiC and Al_2_O_3_ reinforcements, B_4_C possesses numerous advantages, specifically a density (2.51 g·cm^−3^) [4] that is significantly lower than that of SiC or Al_2_O_3_, an extremely high hardness (HV = 30 GPa) and wear resistance, a remarkable chemical inertness [4,5,6,7] and a special neutron absorption capacity [8]. These features make B_4_C an excellent reinforcement for high performance MMCs. Its applications include hard disc substrates, brakes with a high wear resistance and armor plates with a high ballistic performance [9,10]. In recent years, due to the special capturing neutron ability of isotope B^10^, Al-B_4_C MMCs have been increasingly used as neutron shielding materials when fabricating storage containers for spent nuclear fuels in the nuclear industry [11,12,13,14].

One of the main challenges using Al-based MMCs is the influence of the reinforcement particles on the corrosion resistance [15,16,17,18,19]. Because adding reinforcement particles interrupts the continuity of the aluminum matrix and its protective surface oxide films, the number of sites where corrosion could be initiated increases, making the composite more susceptible to corrosion [20,21]. Singh *et al.* [22] studied the influence of SiC particles addition on the corrosion behavior of 2014 Al-Cu alloy in 3.5 wt.% NaCl solution. They found that addition of 25 wt.% SiC_p_ to base alloy decreases corrosion resistances considerably. Zhu and Hihara [23] investigated the influence of alumina fiber on the corrosion initiation and propagation of the Al-2 wt.% Cu-T6 metal matrix composite. Results show that the MMC exhibited inferior corrosion resistance as compared to its monolithic matrix alloy. Bhat *et al*. [24] investigated the corrosion behavior of the 6061 Al-SiCp composite and its base alloy in seawater using the potentiodynamic polarization technique. It was found that the composite corroded faster than its base alloy and that composite corrosion was mainly confined to the interface as opposed to the uniform corrosion observed for the base alloy. Sun *et al.* [25] also studied the corrosion behavior of 6061 Al-SiCp MMCs in a NaCl solution. With the observation that the pitting degree rose with an increasing SiC content, it is presumed that the pitting corrosion depends on the local SiC distribution and the surface film integrity. Roepstorff *et al.* [26] reported that the corrosion resistance of metal matrix composites can be affected by three processes: (1) galvanic coupling of the metal and reinforcement; (2) crevice attack at the metal/reinforcement interface; and (3) preferred localized attack on the possible reaction products between the metal and the ceramic.

In contrast to the many research works dedicated to the corrosion behavior of Al-SiC composites, few studies have focused on the corrosion of Al-B_4_C composites. Ding and Hihara [27] investigated the effect of B_4_C particles on the corrosion behavior of 6092-T6 Al MMCs with 20 vol.% B_4_C in a 0.5 M Na_2_SO_4_ solution at room temperature. Corrosion initiation and propagation are related to the formation of microcrevices, the localized acidification and alkalization of the solution, and to aluminum containing amphoteric oxides. Katkar *et al.* [28] evaluated the effect of the reinforced B_4_C particle content in AA6061 on the formation of a passive film in sea water. They reported that the passive film formed on B_4_C particle-reinforced AA6061 alloy because there was a shift in the corrosion potential toward the positive direction compared to the base alloy. In our previous study [29], the Al-B_4_C composite was less corrosion resistant than the base alloy in the NaCl, K_2_SO_4_ and H_3_BO_3_ solutions. In another study [30], the B_4_C particles exhibited a cathodic character relative to the aluminum alloy in the K_2_SO_4_ solution, meaning that the B_4_C particles could form galvanic couples with the peripheral aluminum matrix in the composite.

To have a deep understanding of the corrosion behavior and corrosion mechanism of AA1100-B_4_C metal matrix composites in a 3.5 wt.% NaCl solution, the present study was carried out in open-to-air and deoxygenated conditions. Electrochemical techniques, including potentiodynamic polarization (PDP), electrochemical impedance spectroscopy (EIS) and zero resistance ammetry (ZRA) were used. Besides, the effect of the B_4_C particle volume fraction on the corrosion behavior of Al-B_4_C composites was investigated, and the surface morphology of the composite before and after corrosion was characterized using an optical stereoscope and a scanning electron microscope (SEM).

## 2. Experimental Procedures

### 2.1. Preparation of Samples and Electrolytes

The investigated composites were AA1100-16 vol.% B_4_C and AA1100-30 vol.%. Both composites were supplied by Rio Tinto Alcan (Saguenay, QC, Canada) via an ingot metallurgy route [3,9]. The average particle size of boron carbide in the composites is 17 μm and the matrix is a standard AA1100 aluminum alloy except the Ti content. Approximately 1.0–2.5 wt.% titanium was added to both Al-B_4_C composites during the composite fabrication process to reduce the interfacial reactions between the B_4_C and the liquid aluminum [31]. The DC cast ingots were preheated and hot-rolled with multi-passes of cross-rolling to the final 4.3 mm thick sheets. To study the effect of B_4_C particles on the corrosion behavior of the composite, an AA1100 alloy without B_4_C was used as the base alloy. The chemical composition of the AA1100 base alloy is listed in Table 1.

**Table 1:** Chemical composition of the AA1100 base alloy.

Elements	Fe	Si	Cu	Mn	Mg	Zn	Al
Composition (wt.%)	0.15	0.10	0.05	0.02	0.001	0.01	Bal.

Samples were cut into small pieces (20 mm by 20 mm) and unless otherwise stated, sanded with a 3M Scotch-Brite™ (3M, Saint Paul, MN, USA) MMM69412 surface conditioning disc (5 inches in diameter, extra-fine surface finish) before being degreased with acetone and rinsed with nanopure water (15.2 MΩ·cm). Finally, all specimens were dried with clean compressed air. Analytical reagent grade NaCl was used to obtain the 3.5 wt.% NaCl electrolyte.

### 2.2. Electrochemical Measurements

The potentiostat employed in the present study was a Reference 600 instrument (Gamry Instruments, Warminster, PA, USA). The electrochemical investigations were performed using a 300 cm^3^-EG&G PAR flat cell (London Scientific, London, ON, Canada) with an Ag/AgCl electrode (4M KCl as filled solution) as the reference electrode and a platinum mesh as the counter electrode (CE). All potentials given in this article are referred to the Ag/AgCl electrode. The corrosion cell had a 1-cm^2^ orifice as the working surface. Magnetic stirring was employed at the bottom of the cell to increase the mass transfer at the electrode surface.

The potentiodynamic polarization tests were performed in open-to-air and deoxygenated conditions. The deoxygenation process began 1 h before the measurements by purging argon into the solution and continued until the end of the experiment. A potential scan was taken from −250 mV below the E_ocp_ to the potential at which a 1 mA·cm^−2^ current density was recorded at a scan rate of 1 mV·s^−1^. The EIS curves were obtained by applying a sinusoidal *perturbation* voltage of 10 mV *rms* around the E_ocp_ in the 100 kHz to 10 mHz frequency range. The detailed polarization and impedance measurements were described in a previous study [15]. Prior to the polarization and impedance tests, all samples were immersed in the 3.5 wt.% NaCl solution for one hour to ensure a steady open circuit potential (E_ocp_). During the galvanic corrosion test, the variations in the galvanic current and potential of the B_4_C wafer and the AA1100 base alloy were recorded continuously for 24 h. In all cases, the tests were duplicated to ensure the reproducibility of the results.

### 2.3. Metallographic Examination

The composite surface morphology was characterized with an optical stereoscope and a scanning electron microscope (SEM, Hitachi SU-70, Hitachi Instruments, Schaumburg, IL, USA) equipped with an energy dispersive spectrometer (EDS). To understand the surface morphology of the composite before and after corrosion, the samples used for the metallographic analysis were polished to a 0.05 μm fine finish.

## 3. Results and Discussion

### 3.1. Microstructure of Al-B_4_C Composites

**Figure 1:**
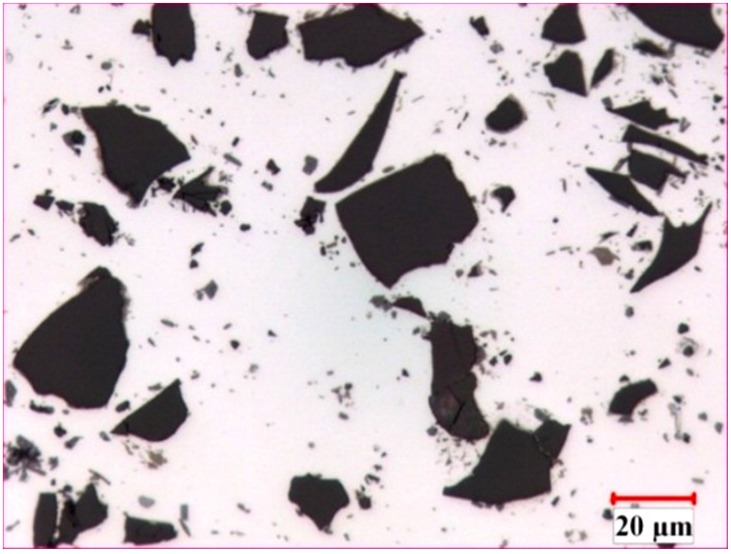
Optical micrograph of hot-rolled AA1100-16 vol.% B_4_C composites.

**Figure 2:**
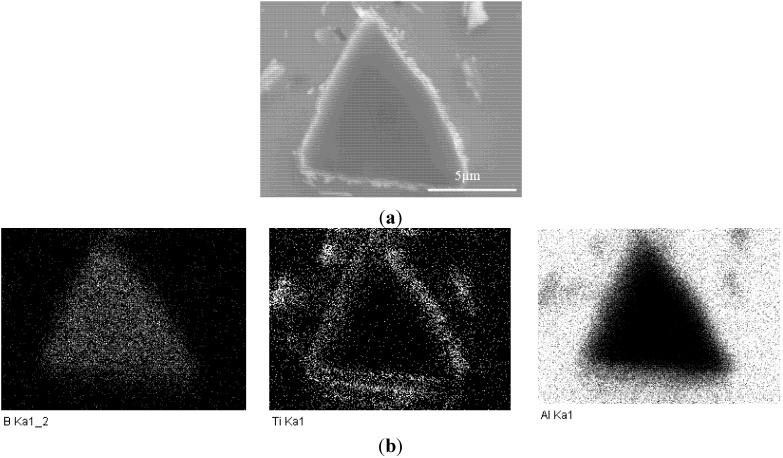
(**a**) SEM image of one B_4_C particle and (**b**) X-ray elemental mapping of (**a**) showing that B_4_C particles are protected by an *in-situ* TiB_2_ layer.

The microstructure of the AA1100-16 vol.% B_4_C composite is illustrated in Figure 1. In general, the B_4_C particles were distributed uniformly in the Al matrix, and two common reaction-induced intermetallic phases were observed in the composite and randomly dispersed in the Al matrix: AlB_2_ (brown, block-like phase) and Al_3_BC (grey phase) [32]. When fabricating the Al-B_4_C composites, the liquid aluminum reacted with B_4_C particles and produced AlB_2_ and Al_3_BC intermetallic particles [3,9]. To limit the reaction between the B_4_C particles and the liquid aluminum, 1.0~2.5 wt.% Ti was added to the composites. Afterward, a thin but dense TiB_2_ layer (a third reaction product) was formed *in situ* at the Al/B_4_C interfaces, isolating the B_4_C particles from the liquid aluminum [9,12]. Consequently, all B_4_C surfaces were surrounded with a TiB_2_ layer, as observed from the SEM micrographs and the X-ray elemental map in Figure 2. The microstructure of the AA1100-30 vol.% B_4_C composite was very similar to the AA1100-16 vol.% B_4_C composite, except for the increased B_4_C amount.

### 3.2. Corrosion Behavior of Al-B_4_C Composites

#### 3.2.1. Potentiodynamic Polarization

**Figure 3:**
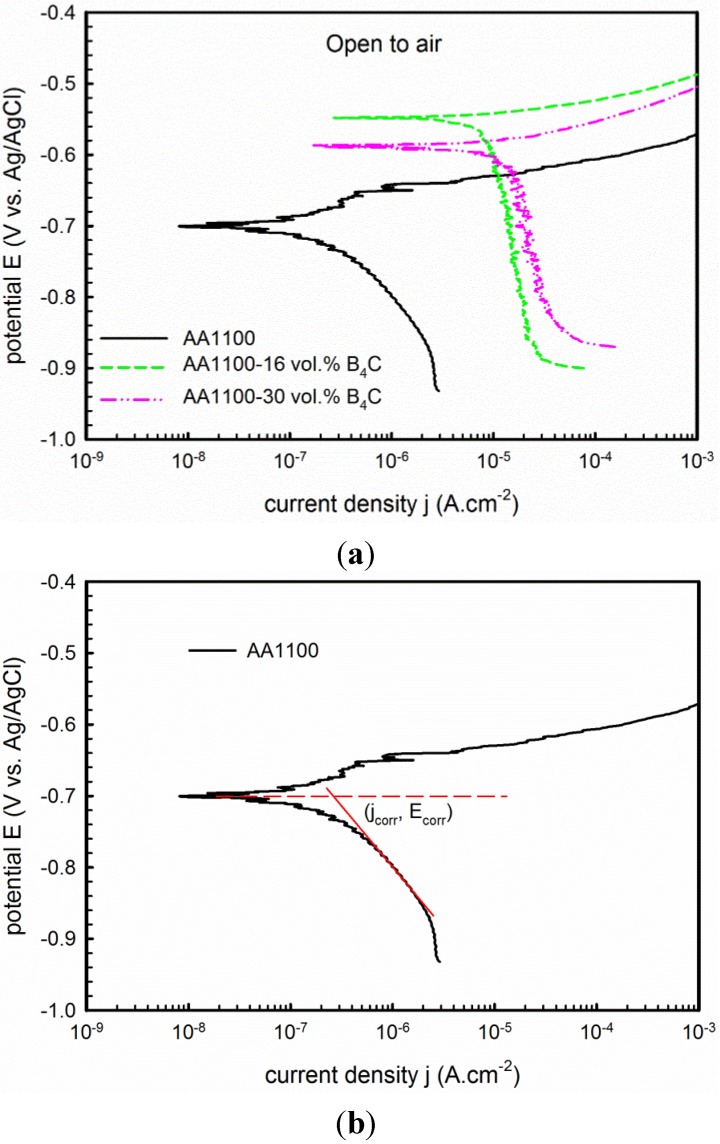
(**a**) Potentiodynamic polarization curve recorded on AA1100, AA1100-16 vol.% B_4_C and AA1100-30 vol.% B_4_C in 3.5 wt.% NaCl in open to air condition; (**b**) Plot showing the j_corr_ was obtained at the intersection point of extrapolation of the cathodic polarization branch and the E_corr_ horizontal line.

The electrochemical behavior of Al-B_4_C composite and effect of B_4_C particle content on corrosion were investigated using potentiodynamic polarization and electrochemical impedance spectroscopy (EIS). Figure 3a displays polarization curves of the composite with different B_4_C contents. The corrosion current density (j_corr_) and corrosion potential (E_corr_) are obtained at the intersection point of extrapolation of the cathodic polarization branch and the E_corr_ horizontal line, which is shown in the Figure 3b. As you will notice from Figure 3a, it is difficult to find a linear region near E_corr_ on the anodic polarization branch of the AA1100 base alloy. Besides, the cathodic branch shows a long and defined linear behavior for over 100 mV; therefore, in this case, as suggested by McCafferty [33], the extrapolation of the cathodic branch method was used. All fittings were done at the liner part of cathodic branch for over 50 mV. The corrosion current density, corrosion potential and cathodic Tafel slopes values are summarized in Table 2.

It shows that the j_corr_ increases from 0.35 to 11.21 μA·cm^−2^ when B_4_C volume fraction increases from 0 to 30 vol.%, suggesting that the corrosion resistance of the composites decreases significantly when increasing the B_4_C volume fraction. The corrosion potential shifts in the positive direction, but the shift does not continue when increasing the B_4_C content. According to the mixed potential theory [28], the potential of the composite is expected to shift in the noble direction when increasing the B_4_C level in the composite. However, increasing the B_4_C volume fraction in the composite increases the discontinuity of the protective surface oxide films, making the Al-30 vol.% B_4_C composite more vulnerable to the chloride ions and generating a less noble potential.

**Table 2:** Electrochemical parameters derived from polarization curves.

Materials	Open to Air			Argon Deaerated		
	E_corr_ (V_Ag/AgCl_)	j_corr_ (μA·cm^−2^)	βc (V/decade)	E_corr_ (V_Ag/AgCl_)	j_corr_ (μA·cm^−2^)	bc (V/decade)
AA1100	−0.70	0.35	0.18	−0.93	0.33	0.16
AA1100-16 vol.% B_4_C	−0.55	8.39	0.71	−0.94	0.99	0.14
AA1100-30 vol.% B_4_C	−0.59	11.21	0.51	−0.93	2.00	0.12

The anodic part of the polarization curve of the base alloy AA1100 reveals an oscillation region beyond which the current density increases quickly, indicating the onset of pitting corrosion. However, for the composite, j_corr_ increases steeply even under low overpotential, implying that pitting is more easily provoked in composites than in the base alloy.

#### 3.2.2. Electrochemical Impedance Spectroscopy (EIS)

**Figure 4:**
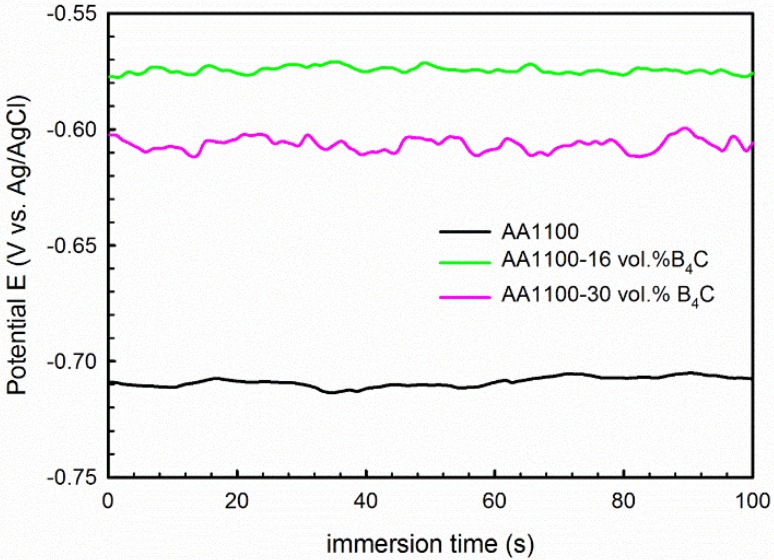
E_ocp_ as a function of time measured 100 seconds before EIS tests, showing the E_ocp_ of AA1100, AA1100-16 vol.% B_4_C and AA1100-30 vol.% B_4_C is stable before EIS tests.

To further confirm the polarization results, EIS measurements were carried out for the composites and base alloy in 3.5 wt.% NaCl solution. Prior to the EIS measurement, the variation of E_ocp_ as a function of time was measured and the graph is shown in Figure 4. It is observed that the E_ocp_ is stabled at −710 ± 5 mV, −575 ± 3 mV and −607 ± 5 mV for AA1100, AA1100-16 vol.% B_4_C and AA1100-30 vol.% B_4_C, respectively.

**Figure 5:**
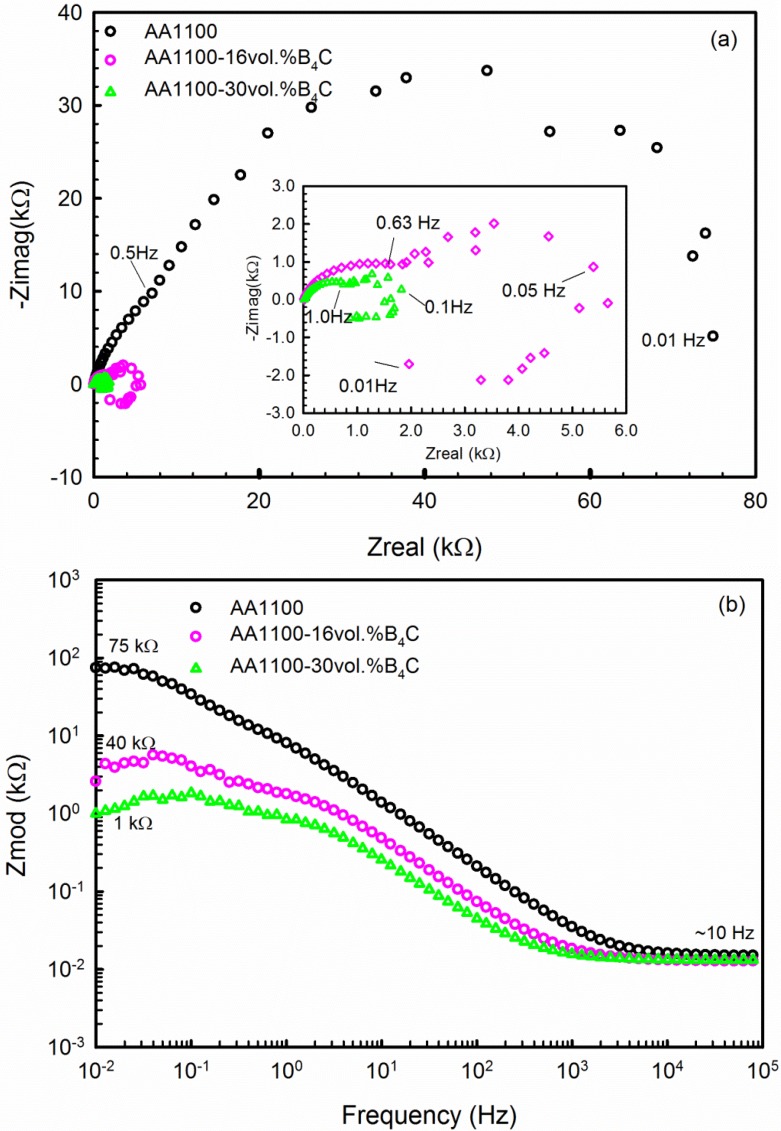
(**a**) Nyquist and (**b**) Bode plot of AA1100, AA1100-16 vol.% B_4_C and AA1100-30 vol.% B_4_C obtained after 1 h immersion in 3.5 wt.% NaCl solution.

The impedance spectra obtained in complex impedance (Nyquist plot) and Bode impedance magnitude are displayed in Figure 5. The EIS spectra show a common characteristic: capacitive semicircles in the high and medium frequency range that are related to the aluminum oxide layer and the electrolyte [28]. The biggest HF semicircle is observed for the base alloy, and the diameter of semicircle decreases when increasing the B_4_C volume fraction in the composite. This observation confirms that incorporating B_4_C particles into AA6061 breaks the continuity of the oxide layer, decreasing its corrosion resistance. The base alloy also has an additional capacitive semicircle in the low frequency range that may be associated with the charge transfer across the alloy–electrolyte interface [34,35]. However, the Al-B_4_C composites show an inductive loop with a reduced charge transfer resistance at the low frequency range, revealing the occurrence of pitting on the composite surface.

The Bode impedance magnitude plot is displayed in Figure 5b. The impedance of the composite at the low frequency range decreases when increasing the B_4_C content. Because the material shows resistive behavior at low frequencies (0.01~0.1 Hz), the impedance at low frequencies could be considered as the resistance. The corrosion resistance of the composite decreases when increasing the B_4_C content, which validates the previous polarization results.

**Figure 6:**
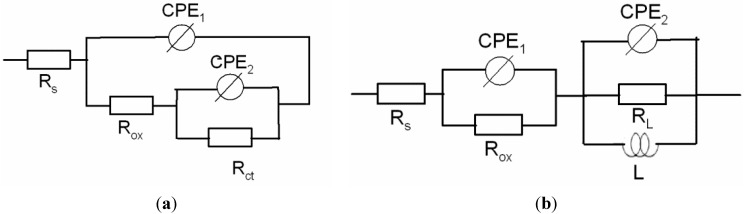
Equivalent circuits proposed for fitting EIS spectra obtained from (**a**) AA 1100 and (**b**) AA1100-16 vol.% B_4_C and AA1100-30 vol.% B_4_C in 3.5% NaCl solution.

**Figure 7:**
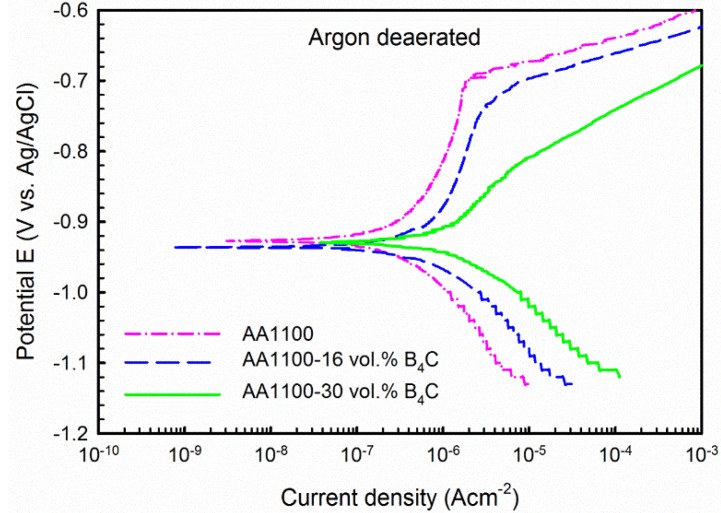
Potentiodynamic polarization curve recorded on AA1100, AA1100-16 vol.% B_4_C and AA1100-30 vol.% B_4_C in 3.5 wt.% NaCl in argon deaerated condition.

Similar EIS spectra were obtained for the B_4_C-reinforced AA6061 alloy in sea water; these data were interpreted using the equivalent circuits shown in Figure 6 [28]. In the first equivalent, R_s_ is the solution resistance, R_ox_ is the aluminum oxide layer resistance and R_ct_ is the charge-transfer resistance of the alloy. CPE_1_ is the capacitance between the electrolyte and the alloy, and CPE_2_ is the capacitance at the interface of the alloy and oxide layer. The equivalent circuit shown in Figure 7b is used to interpret the EIS spectra of the composites. In this equivalent circuit, there are two independent circuits. The first circuit corresponds to the HF capacitive loop that is described with CPE_1_ (double layer capacitance of the oxide layer–electrolyte interface) and R_ox_ (charge-transfer resistance of the oxide layer). The second circuit represents the LF inductive loop; this circuit is described by R_L_ (inductance resistance), L (inductance) and CPE_2_ (capacitance of pit–electrolyte interface).

Because the working electrode deviated from the ideal capacitive behavior due to its surface roughness, heterogeneities, anion adsorption, non-uniform potential, current profile, *etc.* [36], the constant phase element (CPE) was employed to substitute pure capacitances in the equivalent circuits employed.

All parameters derived from the equivalent circuits are summarized in Table 3. The oxide layer resistance R_ox_ decreases from 23.74 to 0.89 kΩ·cm^2^, and the Rct or R_L_ value decreases from 54.26 to 0.96 kΩ·cm^2^ when the B_4_C content increases from 0 to 30 vol.%. These results confirm the polarization results that the corrosion resistance of the composite decreases when increasing the B_4_C content.

**Table 3:** Electrochemical parameters derived from the equivalent circuits in Figure 6.

Materials	R_s_ (Ω cm^2^)	CPE_1_ (μF cm^−2^)	α_1_	R_ox_ (kΩ cm^2^)	CPE_2_ (μF cm^−2^)	α_2_	R_ct_ or R_L_ (kΩ cm^2^)	L (H)
AA1100	14.79	20.83	0.85	23.74	30.93	0.96	54.26	-
Al-16 vol.% B_4_C	12.1	1.0	0.90	1.850	13.0	0.89	4.001	2200
Al-30 vol.% B_4_C	11.0	7.6	0.87	0.898	65.0	0.96	0.960	3200

### 3.3. Corrosion Mechanism Investigation

To study the corrosion mechanism of the composite, polarization tests were conducted in deaerated conditions. The polarization curves are shown in Figure 7 and derived parameters are listed in Table 2. Compared with polarization curves obtained in open-to-air condition, a passive region followed by a well-defined pitting potential at the current density approximately 1 μA·cm^−2^ is observed in the anodic branch for both the base alloy and the composites.

Besides, it is found that three materials have almost the same E_corr_, which means it does not vary with the B_4_C content as observed in open-to-air condition. However, it is more negative than that obtained in open-to-air condition for each material. As seen from Table 2, E_corr_ decreased from −0.70 to −0.93 V_Ag/AgCl_ for the base alloy, from −0.55 to −0.92 V_Ag/AgCl_ for the composite with 16 vol.% B_4_C and from −0.59 to −0.93 V_Ag/AgCl_ for the composite with 30 vol.% B_4_C, respectively. More importantly, the corrosion current density j_corr_ is considerably lower than that obtained in open-to-air condition, *i.e.*, j_corr_ is 0.99 μA·cm^−2^ for the composite with 16 vol.% B_4_C, and 2.00 μA·cm^−2^ for the composite with 30 vol.% B_4_C. This significant difference in current density shows that the corrosion kinetics of the base alloy (AA1100) and the Al-B_4_C composite in the NaCl solution are limited by the oxygen reduction reaction [33].

In addition, the difference on cathodic part of polarization curves was also observed. As seen from Figure 3, the current density remains constant or exhibits very small increases despite the potential increase of the cathodic branch in open-to-air condition. However, this observation was not found in deaerated condition. Similar behavior was observed by Singh *et al.* [30] for 2014-SiC_p_ composites and by Dikici *et al.* [31] for SiO_2_ and Fe/TiO_2_ coated A380-SiC composites in aerated 3.5 wt.% NaCl solution. They believed that this electrochemical behavior is an indicator of the corrosion mechanism, which is controlled by oxygen diffusion.

### 3.4. Galvanic Current Measurement

**Figure 8:**
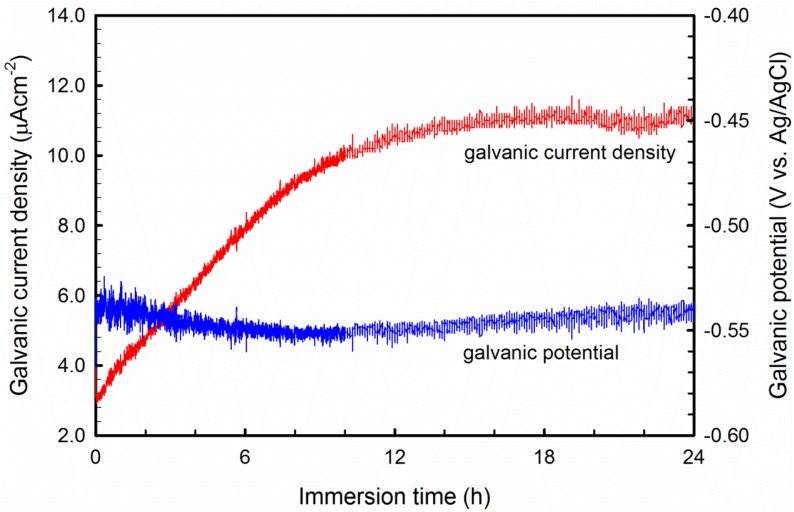
Galvanic current and galvanic potential measured between AA1100 and B_4_C wafer in 3.5 wt.% NaCl solution.

Because Al-B_4_C composites have junctions of two electrochemically dissimilar materials, galvanic corrosion between the Al alloy (matrix) and the reinforcing B_4_C particles may occur, degrading the corrosion resistance. As it is technically difficult to conduct a galvanic coupling test using small B_4_C particles, a hot-pressed 99.5% purity B_4_C wafer produced by Ceradyne, Inc. was used. During the galvanic corrosion test, the base alloy AA1100 was used as working electrode #1 (W1) and the B_4_C wafer was working electrode #2 (W2). A positive galvanic current means that working electrode #1 acts as an anode; otherwise, it acts as a cathode. The measured galvanic current and galvanic potential are shown in Figure 8. The galvanic current during the test is always positive, and the stable galvanic current is approximately 11.2 μA. This result suggests that the Al matrix and B_4_C particles in the composite can form galvanic couples in NaCl solution, and the Al matrix acts as an anode and dissolves. A similar result was obtained by Schneider *et al.* [37], when they studied the galvanic corrosion between pure Nickel and sintered SiC in 3.5 wt.% NaCl solution, *i.e.*, SiC ceramic particles are cathodic sites of the coupling. Abenojar *et al.* [16] also found that the incorporated amorphous Fe/B particles act as cathode and form a strong galvanic couple with the aluminum matrix.

### 3.5. Pitting Morphology

**Figure 9:**
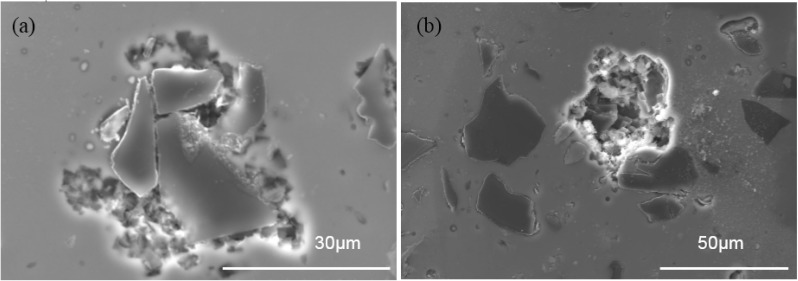
SEM images showing the pitting morphology and initiation places of the AA1100-16 vol.% B_4_C after being polarized to 1 mA·cm^−2^ in 3.5 wt.% NaCl solution: (**a**) at Al–B_4_C interfaces and (**b**) the Al matrix, where intermetallic phases appear.

To identify the initiation sites for the pitting, the AA1100-16 vol.% B_4_C composite sample was polished to 0.05 μm and polarized to 1 mA·cm^−2^. Figure 9 shows that pitting initiated at two sites: (1) the Al–B_4_C interfaces and (2) the sites away from the B_4_C particles in Al matrix where intermetallic phases appeared. Pits at the Al–B_4_C interfaces have an irregular shape, whereas pits in the Al matrix are generally large and hemispherical. Similar types of hemispherical pits were observed on AA5083 in aerated chloride solutions by Abelle *et al.* [38] and Katkar *et al.* [28]. They believed that these pits formed due to the simple detachment of the cathodic precipitates due to gravity. As mentioned in Section 3.1, many small intermetallic particles (AlB_2_ and Al_3_BC) were dispersed in the Al-B_4_C microstructure. Those intermetallic particles might be cathodic relative to the surrounding Al matrix. Due to the galvanic effect, the Al matrix around those intermetallic phases dissolves, detaching the particles.

The formation of pits at the Al–B_4_C interfaces may occur for three reasons: (1) defects existing at the interface between aluminum and B_4_C where Cl^−^ can easily penetrate and attack the Al matrix; (2) the protective TiB_2_ layer at Al/B_4_C interfaces that could be preferentially attacked by chloride ions [39]; (3) the galvanic coupling effect between the Al matrix and the B_4_C particles. Consequently, the Al matrix at the interfaces dissolves, and pits form around B_4_C particles. Once a pit is formed, the local chemical environment is substantially more aggressive than the bulk solution, and therefore the matrix is more severely corroded. Additionally, with increased B_4_C particles in the composite, the cathodic and anodic area ratio (A^C^/A^A^) increases due to the cathodic character of the B_4_C particles; a large A^C^/A^A^ value is detrimental to the Al matrix. Hamdy *et al.* [40] confirmed the occurrence of galvanic and pitting attacks when they studied the corrosion resistance of ALCOA peak-aged AA6092-SiC_17.5p_ composite in 3.5 wt.% NaCl.

**Figure 10:**
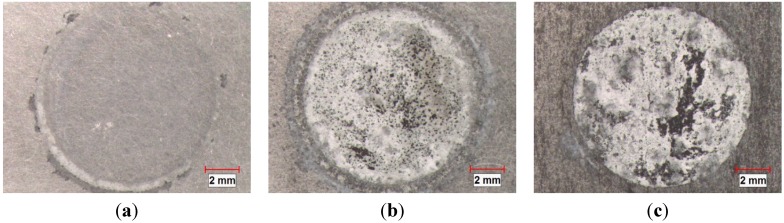
Stereoscope graphs showing the corroded surface morphology of (**a**) AA1100; (**b**) AA1100-16 vol.% B_4_C and (**c**) AA1100-30 vol.% B_4_C immersed in 3.5 wt.% NaCl solution for 10 days.

**Figure 11:**
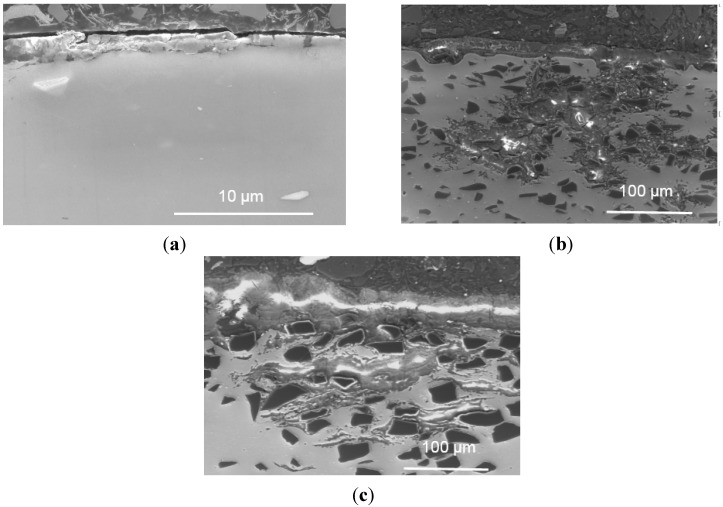
Cross-sectional morphology of (**a**) AA1100; (**b**) AA1100-16 vol.% B_4_C and (**c**) AA1100-30 vol.% B_4_C specimen immersed in 3.5 wt.% NaCl solution for 10 days.

Figure 10 shows the surface appearance of the three materials (the base alloy, AA1100-16 vol.% B_4_C and AA1100-30 vol.% B_4_C) after immersion in 3.5 wt.% NaCl solution for 10 days. The corrosion degree of the test area increases when increasing the B_4_C volume fraction. To evaluate the corrosion of the materials with different B_4_C levels, cross sections from three samples are examined and displayed in Figure 11.

**Figure 12:**
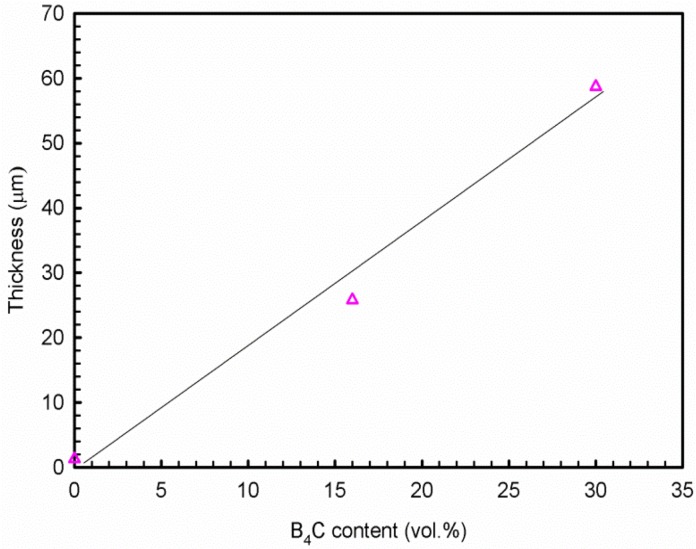
Relationship of corrosion product thickness and B_4_C volume fraction after 10 days exposure in 3.5 wt.% NaCl solution.

Uneven layers of corrosion products formed on the outermost surface of the base alloy and composites. The X-ray elemental maps revealed that these layers consist of aluminum oxide and/or hydroxides. The average corrosion product thickness (the corrosion products in the pits were not included in the calculation of the corroded layer thickness above) is obtained using 20 measurements collected from the SEM images and is presented in Figure 12. It is found that the thickness of the corroded layers increases linearly with the B_4_C content. The thickness of the corroded layer is approximately 1.3 μm in the base alloy and 25.8 μm in the AA1100-16 vol.% B_4_C composite, which is nearly 20 times that of the base alloy. When increasing the B_4_C content to 30 vol.%, the thickness reaches 58.7 μm, which is more than 40 times that of the base alloy. These observations indicate that adding B_4_C particles to the Al alloy can reduce the corrosion resistance in NaCl solution, which accords with the polarization and impedance measurements. Moreover, Figure 11 shows that only the composites suffer severe pitting, which confirms that the Al-B_4_C composite is more sensitive toward pitting than the base alloy in the NaCl solution.

## 4. Conclusions

(1) The polarization and impedance results show that the Al-B_4_C composites are less corrosion-resistant than the base Al alloy and that the corrosion resistance of the composites decreases when increasing the B_4_C particle volume fraction. The cross-sectional images demonstrate that the thickness of corrosion products increases linearly with the B_4_C volume fraction.
(2) Al-B_4_C composites are susceptible to pitting corrosion in the NaCl solution. Two types of pits are observed on the composite surface after polarization in the NaCl solution: (1) pits with an irregular shape that are preferentially initiated at Al/B_4_C interfaces and (2) hemispheric pits that initiate in the Al matrix where the intermetallic particles appeared.
(3) The corrosion of Al-B_4_C composites in 3.5 wt.% NaCl solution is mainly controlled by oxygen reduction in the solution. Moreover, the galvanic couples formed between B_4_C particles and Al matrix is also responsible for the low corrosion resistance.
